# Carrying *Regiella insecticola* Does Not Impose Detectable Life-History Costs on Dominant Chilean *Sitobion avenae* Superclones Under Simulated Heat-Wave Conditions

**DOI:** 10.3390/insects17070730

**Published:** 2026-07-16

**Authors:** Juan Fuentes-Vielma, Daniela A. Sepúlveda, Sebastián I. Martel, Lucía M. Briones, Luis E. Castañeda, Christian C. Figueroa

**Affiliations:** 1 Escuela de Bioquímica, Universidad de Talca, Talca 3460000, Chile; juanfuentesviel99@gmail.com; 2 Research Ring in Pest Insects and Climate Change (PIC2), Santiago 8320000, Chile; simartel@uc.cl (S.I.M.); luis.castaneda@uchile.cl (L.E.C.); 3 Instituto de Ciencias Biológicas, Universidad de Talca, Av. Lircay s/n, Talca 3460000, Chile; lbriones@utalca.cl; 4 Departamento de Ciencias, Facultad de Artes Liberales, Universidad Adolfo Ibáñez, Santiago 8320000, Chile; 5 Núcleo Interdisciplinario de Biología y Genética, Instituto de Ciencias Biomédicas (ICBM), Facultad de Medicina, Universidad de Chile, Santiago 8320000, Chile

**Keywords:** grain aphid, facultative symbiosis, thermal performance, heat wave events, life-history traits, agroecosystems

## Abstract

The intensification of extreme thermal events increases the risk of pest outbreaks and disruptions in agroecosystems, making it crucial to understand how heat waves affect pest performance. This study examined the impact of thermal stress on two dominant aphid genotypes of *Sitobion avenae*, focusing on their symbionts *Regiella insecticola* and *Hamiltonella defensa*. *R. insecticola* is associated with neutrality in performance, while *H. defensa* shows no thermal tolerance. We evaluated symbiont-infected and cured lineages during a heat wave, measuring life-history traits. Key findings include: the *H. defensa* strain incurred increased costs under heat, reducing survival, fecundity, and growth; the effects of *R. insecticola* strain were predominantly neutral and specific to clones; and *Buchnera aphidicola* titers correlated with host performance, with *H. defensa* lineages having higher titers regardless of heat exposure. This suggests that the *R. insecticola* strain present in Chilean genotypes does not contribute to the resilience of certain clones to thermal stress in Chile, whereas the *H. defensa* strain imposes performance costs during heat waves. The results of this research pertain to specific strains of endosymbionts, rather than to all endosymbionts within these taxa. These findings emphasize the need to consider symbiotic relationships and thermal physiology in aphid pest management strategies.

## Simple Summary

As extreme heat events increase, insect pests are more likely to multiply and disrupt farming, raising concerns. Understanding how heat waves affect pest behavior is vital for predicting impacts on crops. For instance, aphids reproduce asexually, are cold-blooded, and can form large groups, sometimes carrying bacteria that help their survival. Our study examined how different clone-symbiont strains combinations in *Sitobion avenae* respond to heat stress, explaining why some perform better in central Chile. We compared two common genotypes with *Regiella insecticola* to a third with *Hamiltonella defensa*, which is not linked to heat tolerance. We tested lines with/without symbionts during a heat wave and tracked traits. Key findings: the strain of *H. defensa* studied increased costs under heat, reducing survival and growth; *R. insecticola* strain effects were neutral and clone-dependent; *B. aphidicola* levels related to performance, with *H. defensa* lines having higher levels, regardless of heat. *R. insecticola* does not aid superclones, while *H. defensa* strain can harm performance during heat. These results emphasize considering symbiosis and heat responses in aphid pest management.

## 1. Introduction

Climate change represents one of the most significant challenges for both biological communities and human welfare [1,2,3]. The gradual increase in average global temperatures (i.e., global warming) is currently accompanied by extreme thermal events (i.e., heat waves) that are becoming more frequent, longer, and more intense. Heat waves are events in which ambient temperatures exceed a specific threshold, defined either in absolute terms or relative to local climatic data [4,5,6]. Mounting evidence highlights the widespread adverse effects of heat waves on agroecosystems and agricultural activities [7,8]. Indeed, heat waves compromise crop performance directly (e.g., by disrupting metabolic functions, affecting phenology, and inducing oxidative stress) and indirectly, by fostering ecological conditions that enhance the success of crop pathogens and insect pests [9,10,11,12]. As extreme thermal events become more frequent and intense, they are expected to affect insect population growth and survival, either by enhancing insect performance or by undermining biological control agents, thereby increasing the likelihood of pest outbreaks [13,14,15]. Hence, understanding how pest species respond to heat waves is essential for understanding outbreak dynamics and designing effective management strategies for future climatic scenarios.

Aphids (Hemiptera: Aphididae) are among the most widespread insect pests in global agriculture, exerting considerable stress on host plants through direct herbivory and the transmission of plant pathogens [16]. Indeed, aphids have been identified as a significant contributor to yield losses attributed to insect pests worldwide [8,17]. Their remarkable invasive success is underpinned by a combination of traits, including cyclical parthenogenesis, wing polyphenism, and phenotypic plasticity, that enable rapid clonal expansion, dispersal, and adaptation to fluctuating environments [16,17]. Moreover, recent research highlights the role of bacterial symbionts in modulating plastic phenotypic responses to environmental stressors [18,19,20]. *Buchnera aphidicola* is an obligate bacterial endosymbiont harbored by all aphid pests, providing essential nutrients to its host [21]. On the other hand, aphids can host other facultative or secondary bacterial symbionts that are not required for survival under benign environmental conditions.

These facultative symbionts can provide context-dependent ecological advantages, including increased resistance to parasitoids and fungal pathogens, broader host-plant use, and, interestingly, tolerance to heat stress [18,20]. As ectotherms, aphid physiology and population dynamics are highly sensitive to temperature, making aphid populations exceptionally responsive to shifts in thermal regimes, including heat waves [15]. Consequently, understanding the interplay among aphid biology, facultative symbiont-mediated plasticity, and temperature regimes becomes critical under the current context of climate change.

Among aphid species, the grain aphid, *Sitobion avenae* (Fabricius), is an important pest species attacking Poaceae cereal systems (wheat, barley, oats, and rye) across temperate regions [22]. This species has been extensively studied in both its native and introduced ranges, particularly in central Chile, where agriculture plays a central role in land use and the economy [23,24]. *S. avenae* was first reported in the 1970s and rapidly established in cereal-growing areas from central to southern Chile [23,25,26,27,28]. Previous work on population genetics showed that the Chilean population of *S. avenae* is dominated by a few highly successful clonal genotypes (i.e., superclones after [28]) which show a reduced genetic diversity compared to their native populations in Europe [23]. Populations of *S. avenae* in central Chile are composed mainly (>80%) by four superclones [23]. Interestingly, the most prevalent (>90%) facultative bacterial endosymbiont in field populations of *S. avenae* in Chile is *Regiella insecticola* [26]. The prevailing genotypes, four in principle (Sa1—Sa4) [23], exhibited spatially variable patterns of association with secondary symbionts: *Regiella insecticola* was reported as the dominant facultative symbiont in central Chile, while *Hamiltonella defensa* was historically more frequent in the south [27]. Although the relative abundance of clonal lineages and endosymbiont composition appear stable, environmental factors such as high temperatures can destabilize the population structure as they reproduce almost exclusively by obligate parthenogenesis [17,29]. Indeed, recent surveys have shown that *R. insecticola* in Chilean populations is now almost fixed among surveyed specimens across the country [26]. However, the potential functional and adaptive significance of this spatial shift in host–symbiont association is unknown.

Atmospheric heat waves in central–southern Chile are on the rise [6]. Despite the key functional role of aphid–symbiont interactions, empirical evidence linking symbiont infection to host performance in the field under heat stress for *S. avenae* remains relatively scarce. In the case of *S. avenae* in Chile, although the distribution and prevalence of *R. insecticola* and *H. defensa* are well documented, it is still unknown if carrying those facultative symbionts could influence survival, fecundity, or developmental performance of their hosts under heat stress conditions, or whether, on the contrary, the observed infection patterns are merely the result of neutral clonal expansion. In this study, we address this subject by evaluating the biological performance of three clonal lineages of *S. avenae* with different infection statuses. Two Chilean lineages, naturally infected with *R. insectcola*, and a third lineage, not present in Chile, infected with *H. defensa*. Because *H. defensa* is known to impose energetic costs on its host under non-stressful conditions, this lineage provided a useful contrast against which to assess the performance of the two Chilean lineages. By exposing these clones to experimental heat wave conditions, such as those observed during late spring–early summer in central Chile, we aim to determine whether the prevalence of *R. insecticola* is related to the success of *S. avenae* superclones in central Chile.

## 2. Materials and Methods

### 2.1. Collection of Aphid Lineages and Endosymbiont Identification

Live specimens of *S. avenae* were collected in wheat fields during spring: across central Chile during November 2016 and from western France during April 2017. All individuals were transported to the laboratory and reared under controlled conditions on a diet of wheat (*Triticum aestivum*, Millán cultivar, INIA-Chile). To ensure parthenogenetic reproduction and the maintenance of each clonal lineage over time, we maintained rearing chambers at 21 °C on a 16:8 h light: dark (L:D) photoperiod (275 ± 40.4 lux), and at ~60% relative humidity (see [30]). The light conditions were instantaneously switched from light to dark and then back to light.

Genotyping of aphid lineages was conducted through routine multiplex PCR amplification of seven microsatellite loci (Sm12, S3.43, S19, S5.L, Sm17, Sm10, and S3.R) following [23] (primers are listed in Table S1). The amplified fragments were analyzed by automated sequencing (ABI 3700 sequencer, Applied Biosystems, Foster City, CA, USA) at Macrogen, Inc. (Seoul, Republic of Korea), and allele sizes were determined one by one using the software GeneMarker^®^ (SoftGenetics, State College, PA, USA). We identified endosymbionts via PCR using specific primers to detect the primary endosymbiont *Buchnera aphidicola*, as well as seven common facultative symbionts, including *Hamiltonella defensa*, *Regiella insecticola*, *Serratia symbiotica*, *Spiroplasma* sp., *Rickettsiella* sp., *Rickettsia*, and *Fukatsuia symbiotica* (for details see [26,27]) (primers are listed in Table S2). The effect of gut microbiota on heat stress in aphids has not been adequately studied, and we did not control for its composition after antibiotic treatment. This is a limitation. Also, we do not exclude the presence of other facultative symbionts at low titers, but preliminary screening did not detect them.

### 2.2. Selection and Establishment of Genotype Lineages

We selected the two currently most widespread and abundant genotypes in central Chile, Sa2 and Sa3 [23], both of which are naturally infected with *R. insecticola* [27]. In the course of our investigation, we were unable to locate live individuals in Chile that were infected with *H. defensa*. Consequently, we incorporated a third lineage of French origin (SaF16), which is intrinsically infected with *H. defensa*, as a control lineage in our study [30]. The inclusion of SaF16 allows us to explore the potential ecological costs or benefits of harboring *R. insecticola*. This may also provide insight into the physiological responses of lineages carrying *H. defensa* that, while currently rare or undetectable in Chilean populations, could remain latent and potentially reemerge under future environmental change.

To evaluate the effects of secondary endosymbionts in aphids under thermal stress, we compared “infected” (E+) and non-infected or “cured” (E−) aphid lineages of the same genotype. We removed endosymbionts using an artificial sucrose-based diet supplemented with antibiotics: ampicillin (100 μg/mL), cefotaxime (100 μg/mL), and gentamicin (400 μg/mL) [31]. We maintained aphid lineages for approximately two years after the antibiotic treatment, and both genotype identity and endosymbiont infection status were reconfirmed before the temperature treatments [30]. All lineages were maintained separately on *T. aestivum* inside rearing chambers under the culture conditions described above (21 °C, 16:8 h L:D, ~60% RH).

### 2.3. Experimental Setup and Temperature Treatments

Synchronized nymphs no older than 24 h, representing each combination of aphid genotype (Sa2, Sa3, and SaF16) and symbiont infection status (E+ and E−), were individually transferred to fresh 10-day-old wheat seedlings (*T. aestivum*, cultivar Millán, INIA-Chile). Plants were grown using a standardized protocol, with three seeds sown in 4 cm-diameter pot filled with 7 g of a 1:1 perlite–vermiculite mixture. Each pot was covered with a perforated plastic bag to preserve the integrity and individuality of the experimental unit while allowing gas exchange. These aphids were considered the first generation (G1).

Temperature treatments were conducted inside a controlled incubator chamber (Pitec, Bioref-38, Santiago, Chile) set to the baseline conditions described above to ensure parthenogenetic reproduction. Over the past decade, during the megadrought [32], central Chile has experienced three to four non-consecutive periods in which maximum temperatures reached up to 34 °C in months such as November (late spring) and December (early summer). Daily temperature data were obtained from the CR2MET v2.5 climate dataset [33].

To simulate the thermal stress associated with the irregular heat wave patterns reported during spring and early summer in central Chile [34], we raised the 21 °C baseline temperature to 34 °C three times per week for 2 h [35] (Monday, Tuesday and Friday), using a heating rate of 0.2 °C/min, totaling ~2 h per trial (hereafter, HW). The heating rate was chosen for technical reasons (equipment capabilities) and does not simulate natural scenarios. This is a limitation. To assess the leaf temperature, five plants were measured three times each using a visual IR thermometer (Fluke VT04) (Control 21 °C: leaf temperature 22.1 ± 0.105 SE; Treatment 34 °C: leaf temperature 34.8 ± 0.182 SE.

During heat treatments, control lineages were kept under baseline conditions (i.e., 21 °C; hereafter Control). The temperature treatments were kept consistent until all measurements were completed, as detailed below. After the temperature treatment, all individuals from HW and Control were maintained under baseline conditions in a single chamber, so the post-trial environment was identical across groups. To ensure the independence of experimental units, pots were randomly repositioned within the chamber every day. Aphids were transferred to fresh 10-day-old wheat seedlings on day 13 post-trial. To examine maternal effects, we collected nymphs born on days 12–13 (>24 h old, second generation, G2) and placed them on fresh 10-day-old *T. aestivum* seedlings. The thermal treatment was then repeated following the same schedule. The experiment concluded when G2 individuals reached adulthood. The total sample size per treatment level was 18 individuals. For SaF16-E+, however, 36 individuals were initially included to account for the high mortality observed in preliminary trials of this lineage. Overall, the experiment comprised 378 individuals.

### 2.4. Life-History Traits

To assess how the experimental temperature treatment influence aphid performance, we measured four integrative components of life-history: (i) survival and (ii) adult body mass, both across two successive generations (G1–G2); (iii) fecundity; and (iv) intrinsic population growth rate (*i*)—a demographic parameter that integrates survival, development time, and fecundity into a single estimate of population growth potential—both measured only in G1. Each nymph was monitored daily from the beginning of the treatment until twice the number of days since its first reproduction event (i.e., laying nymphs). Following its first reproduction, each aphid was observed daily for a number of days equal to its pre-reproductive period, and we recorded mortality and the number of offspring [36]. Considering the differential energetic demands, individuals that developed into winged morphs were excluded from the analyses. Finally, *r_m_* was calculated using the equation proposed by Wyatt and White [37]:

*r_m_* = 0.738 × (*ln M_d_*)/*T_d_*

where *T_d_* represents the number of days until first reproduction, and *M_d_* is the total number of offspring produced during that same period. Body mass (in mg) was recorded for each G1 and G2 individual at the onset of reproduction using an analytical microbalance (Mettler Toledo, XS3DU, Greifensee, Switzerland). We did not record the frequency of winged individuals, so we cannot eliminate potential bias. This represents a limitation.

### 2.5. DNA Extraction and Endosymbiont Quantification by qPCR

Environmental factors, such as high temperatures, can modify the abundance of obligate and facultative endosymbionts, leading to changes in symbiont abundance and significant performance costs for aphids [38]. However, aphid performance can also be modulated by the facultative endosymbiont strain [39].

Hence, we first assessed intraspecific variation among facultative endosymbionts by sequencing four housekeeping genes (*accD*, *gyrB*, *recJ*, and *rpoS*) as described by [39]. The sequencing was performed by the Sanger method at Australomics (Valdivia, Chile), and the resulting sequences (available in NCBI, Table S3) were aligned, concatenated, and analyzed using Geneious Prime^®^ v2024.0.7. The sequencing showed that all Sa2 and Sa3 aphids harbored the same *R. insecticola* strain. Additionally, the presence of the APSE bacteriophage, a double-stranded DNA phage associated with protection against parasitoid infections in the pea aphid [40], was corroborated in aphids of the genotype SaF16 by amplifying three genes involved in key viral functions: lysis (P3), host cell entry (P35), and regulation (P51), as described by Degnan and Moran [41] (available in NCBI, Table S3).

Second, the titers of obligate and facultative endosymbionts were determined in 7-day-old aphids from both Control and HW treatments, corresponding to the first reproductive event, on average. Sampled individuals were preserved in 95% ethanol, and DNA was extracted from each aphid using the salting-out protocol described by Sunnucks and Hales [42]. Endosymbiont quantification was performed with three biological replicates for each combination of treatment (Control and HW), clonal lineage (Sa2, Sa3, and SaF16), and secondary endosymbiont infection status (E+ or E−). Thus, each biological replicate consisted of an equimolar pool of DNA from three aphids that shared the same treatment, lineage, and infection status. The DNA extracted from each aphid was adjusted to a concentration of 50 ng µL^−1^ before pooling. Quantification of the relative abundance of *B. aphidicola*, *R. insecticola*, and *H. defensa* in both Control and HW aphids was performed via quantitative PCR (qPCR), using symbiont-specific primers described in [30] (Table S4). Elongation factor 1-α (EF-1α) was used as the reference gene. Amplifications were carried out using a Stratagene Mx3000P thermocycler (Agilent Technologies, Santa Clara, CA, USA) and the Takyon™ ROX SYBR 2X Master Mix dTTP blue kit (Applied Biosystems, CA, USA). Each 20 μL reaction mixture contained 8.2 μL of ultrapure H_2_O, 10.0 µL of Takyon 2X Master Mix, 0.4 µL of each primer (forward and reverse, 10 pmol), and 1.0 µL of template DNA. The qPCR cycling conditions were as follows: an initial denaturation at 94 °C for 10 min; followed by 35 cycles of 94 °C for 30 s, 60 °C for 30 s, and 72 °C for 1 min. Before qPCR analysis, the symbiont status of the studied genotypes (infected and cured) was assessed after measuring life-history traits.

### 2.6. Statistical Analyses

Given the limited replication at each treatment level (n = 18), due to the logistical complexity of the experiment, and the multifactorial nature of the design, which included 18 treatment combinations and several potentially interacting factors, we adopted a two-step analytical approach to assess the effects of temperature on the life-history traits of *S. avenae* (survival, body mass, fecundity, and *r_m_*). First, using only G1 individuals under control conditions (21 °C), we tested the effects of genotype and endosymbiont status, including their interaction, to determine whether control data could be pooled as a common baseline or needed to be explicitly accounted for in subsequent analyses. Second, when the first-step analyses (Control-G1) indicated that control data should be accounted (*p* < 0.05 in any source of variation), we fitted full-factorial models (linear models, LM; or generalized linear models, GLM) to the experimental data, including *genotype* (Sa2, Sa3, SaF16), *symbiont* status (E−, E+), *generation* (G1, G2; when available), and *treatment* (Control, HW) as fixed factors. Only experimental individuals included in the heat wave treatment were considered in the analysis; in other cases, interaction terms were included up to the highest order supported by the data (e.g., genotype × symbiont × generation × treatment). Models were specified according to the distribution of each response variable: survival (0/1; binomial GLM), body mass (continuous; Gaussian LM), fecundity (counts; Poisson GLM), and *r_m_* (continuous; Gaussian LM). When the highest-order interaction was not significant, models were simplified by sequentially removing non-significant interaction terms, resulting in reduced models (e.g., from genotype × symbiont × generation × treatment to genotype × symbiont). Due to unbalanced replication across treatment levels (e.g., by differential survival), we evaluated global effects of model terms using Type-III tests, based on likelihood-ratio χ^2^ test for GLMs and F-tests for Gaussian LMs. We conducted post hoc comparisons using estimated marginal means (EMMs) on the response scale, with pairwise Tukey adjustments to compare experimental levels. When interactions were significant, we conducted targeted pairwise simple-effects contrasts (e.g., genotype within symbiont × treatment; symbiont within genotype × treatment). We visually checked model assumptions using standard residual diagnostics. Regarding the relative abundance of *B. aphidicola* and facultative symbionts, given that the primary objective was to evaluate the effects of the presence of secondary symbiont and heat wave treatment on *B. aphidicola* titer, each aphid lineage was analyzed independently by comparing E+ and E− individuals using traditional linear models. All analyses were performed in the R statistical environment [43] using the stats (base R), car [44], and emmeans packages [45].

To evaluate the sensitivity of the analyses, we performed simulation-based post hoc power analyses for the major planned contrasts. The experimental design included multiple factors and interactions, generalized response distributions, and in some cases unbalanced replication across treatment combinations; thus, we used parametric simulations. For each response variable, 1000 datasets were simulated from the fitted models while preserving the observed experimental structure. The same model was then refitted to each simulated dataset, and the corresponding estimated marginal means contrasts were recalculated. Power was estimated as the proportion of simulations in which the target contrast was statistically significant at α = 0.05, using the same post hoc comparison structure as in the main analysis.

Post hoc comparisons were performed using two-sided tests with α = 0.05, based on Tukey-adjusted pairwise or simple-effects contrasts. Figures display raw data as jittered points, together with estimated means and standard errors (±SE). Survival is presented as predicted survival probability, fecundity as expected counts from the Poisson model, and body mass and *r_m_* as expected means from Gaussian models. Significance letters or asterisks were not included in the figures because the large number of pairwise contrasts would reduce readability. Complete statistical comparisons are reported in the corresponding Supplementary table.

## 3. Results

Descriptive summaries of evaluated traits across genotypes, facultative symbiont status, treatments, and generations are provided in Table S5. In G1 controls (21 °C), survival and adult mass did not differ by genotype, symbiont, or interaction (Table 1), so control individuals were not analyzed further for these traits. Therefore, only individuals exposed to the heat wave treatment were considered in subsequent analyses of these traits. In contrast, fecundity and *r_m_* showed significant genotype × symbiont interactions (χ^2^(2) = 18.859, *p* < 0.001; F_2,94_ = 3.696, *p* = 0.028, respectively; Table 1), and data from both control and heat wave treatments were retained for further analyses.

**Figure 1:**
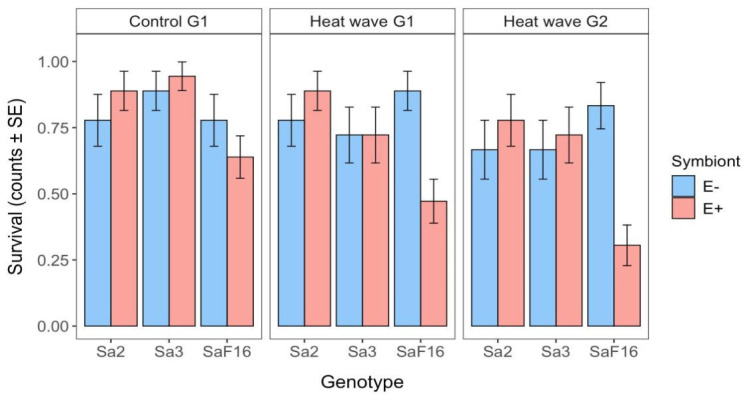
Survival probabilities (±SE) of *S. avenae* genotypes (Sa2, Sa3, and SaF16) carrying (E+) or cured of (E−) facultative symbionts across two successive generations (G1 and G2) after heat wave treatment (n = 18; n = 36 for SaF16 E+).

In full-factorial models, survival exhibited a significant genotype × symbiont interaction (χ^2^(2) = 7.88, *p* = 0.019; Tables S6 and S7, Figure 1). Under the heat wave regime, survival of SaF16/E+ dropped sharply—and significantly (*p* < 0.05)—versus every other genotype × symbiont combination, whereas SaF16-E matched Sa2 and Sa3 across all combinations (see Table S2).

**Figure 2:**
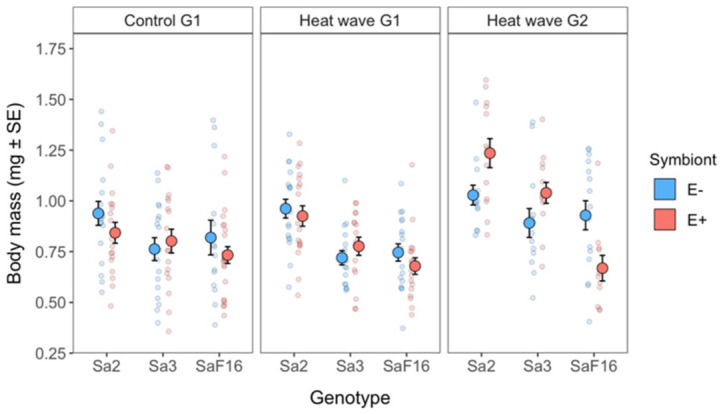
Adult body mass of *S. avenae* genotypes (Sa2, Sa3, and SaF16) carrying (E+) or cured of (E−) facultative symbionts across two successive generations (G1 and G2) after heat wave treatment. Each small point represents an individual aphid. Larger symbols and lines depict estimated means (±SE) (n range = 12–18).

Adult body mass showed a genotype × symbiont × generation interaction in the full model (F_2,171_ = 4.37, *p* = 0.014; Table S3). This indicates that after the heat wave treatment, the effect of the symbiont on body mass varied across the factor generation and depended on the considered genotype (Figure 2, Tables S8 and S9).

**Figure 3:**
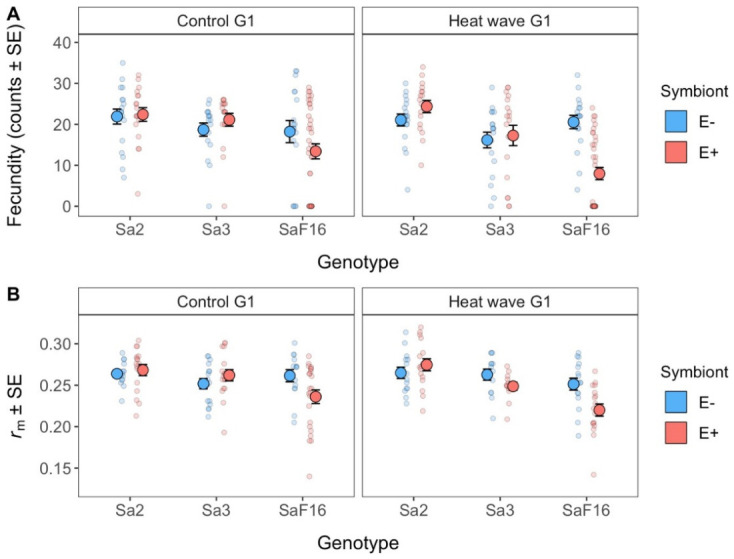
Reproductive performance of *S. avenae* genotypes (Sa2, Sa3, and SaF16) carrying (E+) or cured of (E−) facultative symbionts across two successive generations (G1 and G2) after heat wave treatment. (**A**) Fecundity (number of produced offspring; n = 18; n = 36 for SaF16 E+). (**B**) Intrinsic population growth rate (*r_m_*). Each small point represents an individual aphid. Larger symbols and lines depict estimated means (±SE; n range = 12–18; n = 23 for SaF16 E+ control).

Fecundity revealed a three-way interaction, genotype × symbiont × treatment (χ^2^ (2) = 29.17, *p* < 0.001; Table S10) mainly pointing towards SaF16/E+ produce fewer nymphs than SaF16/E−, in both Control and—fewer in—HW treatments (*p* < 0.001); and, simultaneously, differential effects for targeted pairwise simple-effects contrasts, depending on the combination of factor levels (Figure 3A, Table S11). As expected, the intrinsic population growth rate largely mirrored fecundity, in that the *r_m_* of SaF16/E+ was significantly lower than all other combination genotype × symbiont combinations, regardless of the treatment (Control or HW; Figure 3B, Tables S12 and S13). Altogether, SaF16/E+ under the HW regime showed the lowest survival, body mass, fecundity, and *r_m_*, compared with Sa2 and Sa3 (Figure 1, Figure 2 and Figure 3).

**Figure 4:**
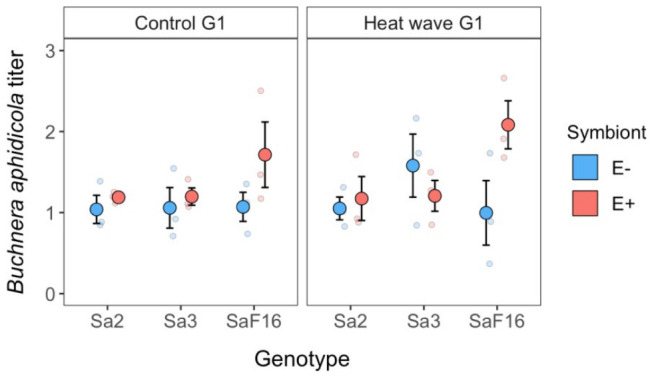
Relative titer of the obligate symbiont *B. aphidicola* in *S. avenae* genotypes (Sa2, a3, and SaF16) carrying (E+) or cured of (E−) facultative symbionts under control (C) and heat wave (HW) treatments. Values represent estimated means (±SE) derived from lineage-specific analyses.

The abundance analysis showed that the *B. aphidicola* titer increased only in SaF16-E+, independently of the level of heat treatment (Control or HW). In contrast, no comparable changes were observed in Sa2 or Sa3 (Figure 4, Table S14). On the other hand, facultative symbionts *H. defensa* and *R. insecticola* did not vary between the Control and HW regimes (*p* > 0.05) (Table S15, Figures S1–S3).

The simulation-based post hoc power analyses indicated that statistical power was strongly dependent on the trait and contrast considered. Power was high for the main SaF16-associated differences in survival and fecundity, suggesting that the study had adequate sensitivity to detect the strongest observed effects involving the *Hamiltonella*-associated lineage. However, non-significant genotype-specific contrasts, particularly for body mass and *r_m_*, should be interpreted cautiously, as they may reflect limited sensitivity to detect small or moderate differences rather than clear evidence of no effect (Table S16).

## 4. Discussion

Three main insights can be deduced from our results. First, carrying *H. defensa* imposes a clear cost that intensifies under a heat wave regime. The SaF16 lineage performed poorly in terms of survival and overall performance only when infected (E+); conversely, once cured (E−), its performance converged with that of Sa2 and Sa3. Moreover, survival, as an integrative performance indicator, dropped sharply in SaF16/E+ after the exposure to HW treatment (Figure 1), entailing lower fecundity and a reduced intrinsic population growth rate, *r_m_* (Figure 3). As can be perceived in Figure 1, Figure 2 and Figure 3, the direction and magnitude of the differential performance of SaF16/E+ compared to SaF16/E− clearly indicate the effect of carrying the symbiont (i.e., *H. defensa*) rather than the aphid’s genotype. Second, carrying *R. insecticola* appears to have little impact on the performance of the studied *S. avenae* lineages when exposed to HW treatment (Figure 1, Figure 2 and Figure 3). Genotype Sa2 consistently, although not statistically significant, exceeds Sa3 in body mass, fecundity, and *r_m_* (Figure 2 and Figure 3).

This is critical, as small advantages can scale up in the field: slightly lower heat tolerance or, conversely, higher physiological demands in Sa3 may translate into reduced population growth and even competitive exclusion of Sa3 by Sa2 (see [29]). Despite previous studies reporting that *R. insecticola* can depress performance [46,47] and reduce survival after heat shock events [48], the sign and the magnitude of the effect seem to rely on the host genotype and the ecological context [49,50]. Moreover, differences in performance among lineages are consistent with the idea that the realized effects of facultative symbionts depend on the specific genotype–symbiont and even symbiont strain associations [39,51]. Notably, body mass did not map one-to-one onto population growth, indicating a partial decoupling between body mass and *r_m_* (Tables S8 and S12). Also, following the heat wave treatment, the SaF16–E+ association failed to show the weight gains observed in the other combinations (Table S8). Third, the obligate symbiont *B. aphidicola* mirrored these outcomes as its titer was higher in SaF16 when carrying *H. defensa* (SaF16/E+), in both levels of treatment Control and HW treatments. Indeed, *B. aphidicola*, when SaF16 was infected with *H. defensa*, exhibited a marked rise relative to E− (Figure 4). This *B. aphidicola* decoupling in SaF16/E+ is consistent with disrupted host homeostasis when *H. defensa* is present, possibly representing an elevated energetic demand, and plausibly explaining the constrained fecundity and *r_m_* of this genotype × symbiont combination [52]. Taken together, these results suggest a genotype × symbiont × environment interaction that helps explain the dominance of Sa2, as observed in recent field surveys. In contrast, a superficially similar competitor carrying the same secondary symbiont (Sa3) remains comparatively less prevalent [27]. Mechanistically, our data point to (i) energy allocation trade-offs, depicted by the differential performance of all lineages when carrying facultative symbionts compared with the cured clones; and (ii) a compensatory organismal response, carried over into G2 likely mediated by maternal effects, evidenced, for instance, by a modest weight gain (Figure 2) or by rebalancing the titer of the obligate symbiont (Figure 4). Indeed, the significant increase in the relative abundance of *B. aphidicola* when *H. defensa* is present—observed in SaF16-E+—accompanied by declines in *survival* and *r_m_* is consistent with costly symbiont proliferation or a breakdown in host control over facultative symbionts. However, *R. insecticola* loads did not affect the *B. aphidicola* titers either in Sa2 or Sa3, suggesting low incidence in its regulation (Figure 4). These observations reinforce the idea that the beneficial effects of facultative symbionts lie on a continuum that depends on genotype–symbiont interaction, and, in this case, thermal adaptation of each lineage [53,54]. On the other hand, *B. aphidicola*, which supplies essential amino acids, is central to the energetic balance, and both its titer and function are temperature sensitive [55]. In this vein, it seems that carrying *H. defensa* disrupts *B. aphidicola* homeostatic titer, increasing metabolic demands [52], and in the case of SaF16-E+, the energy allocated to maintenance may be insufficient to control the infection properly. Hosts must then allocate limited resources between self-maintenance (e.g., repairing damage, producing heat-shock proteins) and restraining the proliferation of facultative symbionts, which, in the absence of the ecological conditions that confer benefits, can represent an energetically demanding infection [50,56]. While some thermal perturbations can be transient, with physiological recovery in subsequent benign periods [15,57], our results show that even brief heat wave episodes can unmask substantial symbiont-mediated energetic costs. Moreover, warming elevates insect metabolism, reshapes organismal homeostasis, and alters population growth, thereby intensifying pest infestations [9,53].

Nevertheless, our study also has system-specific limitations. Working with superclones can be a strength as it reduces genetic noise and increases the power to detect symbiont effects. However, it also narrows inference: there are few host–symbiont combinations, competitive exclusion can quickly remove aphid lineages, and natural selection can drive rapid temporal lineage turnover (see [29]). For these reasons, we included the non-local SaF16 lineage. While it reveals clear *H. defensa* costs, some conclusions may not fully translate to the geographic distribution of lineages in central Chile. Moreover, colonies maintained for more than 100 generations under constant laboratory conditions may display a downregulation of plastic responses observed in nature. To address these issues, future work should perform reciprocal facultative symbiont transplants; vary heat wave intensity, duration, and timing; and validate patterns along Chile’s climatic gradient through longitudinal surveys of clonal frequencies, symbiont loads, and parasitoid pressure. Such tests will determine whether the energetic costs detected here persist over time, and whether they predict real-world turnover and persistence. However, the results presented here may represent a good starting point.

Overall, our results suggest that no effect was detected under the tested conditions. Whereas *H. defensa* strain imposed clear performance costs under the experimental heat wave regime, carriage of the *R. insecticola* strain had little effect on *S. avenae* performance under the same conditions. This finding is particularly relevant because, as temperatures rise, the intervals between heat waves may become key windows for aphid outbreaks. More broadly, the geographic and temporal distribution of symbioses may reflect genotype-by-environment trade-offs. Recognizing this contingency is crucial for explaining current distribution patterns and for designing climate-informed aphid management strategies aimed at limiting both economic losses and disease transmission.

Our findings emphasize the role of genotype–symbiont interactions in aphid responses to heat waves. The SaF16 lineage showed the most significant effects, with *H. defensa* strain linked to lower survival, reproduction, body mass, and population growth. In contrast, *R. insecticola* strain had minimal impact on the two main Chilean genotypes.

An important issue to consider is the presence of the specific bacteriophage APSE found in *H. defensa* strain. The costs associated with particular strains of APSE depend on the toxins present in *H. defensa* [40], which include Shiga-like toxin, cytolethal distending toxin, and YD-repeat toxins. This information helps to explain the results obtained from the genotype infected with *H. defensa* in this study. Unfortunately, this represents a limitation in this study, as we did not identify the APSE strain present in the genotype infected with *H. defensa*.

The findings of this study are related to particular strains of endosymbionts found in aphids, rather than the endosymbionts as a whole. Future reciprocal symbiont-transfer experiments are needed to explore the broader implications of these patterns.

## Figures and Tables

**Table 1:** Results of initial analysis of G1 aphids maintained under control conditions (21 °C), testing the effects of genotype, symbiont status, and their interaction on life-history traits. Bold: significant values.

Survival	Effect Source	Likelihood-Ratio Test χ^2^	df	*p*
	genotype	1.053	2	0.591
	symbiont	0.813	1	0.367
	interaction	2.288	2	0.318
	Effect			
Body mass	Source	F test	df	*p*
	genotype	2.422	2	0.093
	symbiont	1.414	1	0.237
	interaction	0.847	2	0.431
Fecundity	Effect source	Likelihood-Ratio test χ^2^	df	*p*
	genotype	7.122	2	**0.028**
	symbiont	0.101	1	0.749
	interaction	18.859	2	**<0.001**
*r_m_*	Effect source	F test	df	*p*
	genotype	0.749	2	0.475
	symbiont	0.175	1	0.675
	interaction	3.696	2	**0.028**

## Data Availability

The data that support the findings of this study are available from the corresponding author upon reasonable request.

## Supplementary Materials

The following supporting information can be downloaded at: https://www.mdpi.com/article/10.3390/insects17070730/s1, Table S1: Microsatellites used to identify multilocus genotypes in *Sitobion avenae* [58,59,60], Table S2: PCR primers used to detect secondary symbionts in *Sitobion avenae* [61,62,63], Table S3: Accession codes of the sequences obtained from facultative endosymbionts, Table S4: Primer sequences used for qPCR [30,64,65], Table S5: Summary of evaluated traits across genotypes, facultative symbiont status, treatments, and generations, Table S6: ANOVA table for survival, Table S7: Pairwise contrasts for survival, Table S8: ANOVA table for body mass, Table S9: Pairwise contrasts for body mass, Table S10: ANOVA table for fecundity, Table S11: Pairwise contrasts for fecundity, Table S12: ANOVA table for Intrinsic population growth rate (*r_m_*), Table S13: Pairwise contrasts for Intrinsic population growth rate (*r_m_*), Table S14: ANOVA table for *Buchnera aphidicola* titer (full model) and individual ANOVA models within genotype, Table S15: ANOVA table for the facultative symbiont titer within genotype, Table S16: Results of the simulation-based post hoc power analyses. Figure S1: Relative titer of *R. insecticola* in genotype Sa2 from *S. avenae* under Control (C) and heat wave (HW) treatments and generations (g1 and g2). Figure S2: Relative titer of *R. insecticola* in genotype Sa3 from *S. avenae* under Control (C) and heat wave (HW) treatments and generations (g1 and g2). Figure S3: Relative titer of *H. defensa* in genotype SaF16 from *S. avenae* under Control (C) and heat wave (HW) treatments and generations (g1 and g2).
