# Supplementary material for: Carrying Regiella insecticola Does Not Impose Detectable Life-History Costs on Dominant Chilean Sitobion avenae Superclones Under Simulated Heat-Wave Conditions
Source: Insects. 2026 Jul 16;17(7):730. doi: 10.3390/insects17070730 (PMC13411810; doi:10.3390/insects17070730)
Supplement: Supplementary file 1 [file insects-17-00730-s001.zip › insects-4324626-supplementary.pdf]

Supplementary Material
Fuentes-Vielma et al.

Table S1. Microsatellites used to identify multilocus genotypes in *Sitobion avenae*

Locus	Forward (5′-3′)	Reverse (5′-3′)	Source
Sm12	CACCATCGCGTTTCATCTTA	ACT CCCAACCTCTGATGAGC	[1]
S3.43	GGCGAGACCCCTTAAAATCC	GAGATACTCTTTTCGTCGTTAAACC	[2]
S19	GCGCATTGTGTAGCGAGC	CAAACATGTTATGTCACAATAC	[2]
S5L	GGACGACTCGTTAGTATAGGTGG	CTATCTCTACCGTTTCGAATCG	[2]
Sm17	TTCTGGCTTCATTCCGGTCG	CGTCGCGTTAGTGAACCGTG	[2]
Sm10	TCTGCT GCATTACTGTTGGC	TCGTCTACTTCGCCGTCA	[3]
S3R	CATCCGAGCGGTGGAATG	CATTTCGTCATCATTTGCTACATG	[2]

Table S2. PCR primers used to detect secondary symbionts in *Sitobion avenae* (extracted from [4])

Primer name	Primer sequence (5'-3)	Specificity	Amplicon size (bp)
16SA1	AGAGTTTGATCMTGGCTCAG	Generic	
Spi1500R	ATCATCAACCCTGCCTTTGG	*Spiroplasma sp*	1500
PAUS16SR	TCGGACGCCATAACACTAGG	*Regiella insecticola*	840
PABS480R	GGTATTCGCATTTATCGCTTC	*Hamiltonella defensa*	480
P136Ric-470R†	TGGGTACCGTCACAGTAATCGA	*Rickettsiella sp*	300
P136F†	GGGCCTTGCGCTCTAGGT	*Rickettsiella sp*	
PAXS F‡	AGTTTGATCATGGCTCAGATTG	*PAXS*	500
PAXS R‡	GCAACACTCTTTGCATTGCT	*PAXS*	
Buch270R	TGCCTTGGTAGGCTATTACTC	*Buchnera*	270
PASS1140R	TTTGAGTTCCCGACTTTATCG	*Serratia symbiotica*	1140
Ric600R	TTTGAAAGCAATTCCGAGGT	*Rickettsia sp*	600

†Developed by [5]

‡Developed by [6]

Table S3. Accession codes of the sequences obtained from facultative endosymbionts, available in NCBI.

Gene	Multilocus genotype	Target	Accession code
rpoS	SaF16	*Hamiltonella defensa*	PZ519234
rpoS	Sa3	*Regiella insecticola*	PZ519235
rpoS	Sa2	*Regiella insecticola*	PZ519236
recJ	SaF16	*Hamiltonella defensa*	PZ519237
recJ	Sa3	*Regiella insecticola*	PZ519238
gyrB	SaF16	*Hamiltonella defensa*	PZ519239
gyrB	Sa3	*Regiella insecticola*	PZ519240
gyrB	Sa2	*Regiella insecticola*	PZ519241

AccD	SaF16	*Hamiltonella defensa*	PZ519242
accD	Sa3	*Regiella insecticola*	PZ519243
AccD	Sa2	*Regiella insecticola*	PZ519244
recJ	Sa2	*Regiella insecticola*	PZ519245
P51	SaF16	APSE	PZ519246
P35	SaF16	APSE	PZ519247
P3	SaF16	APSE	PZ519248

Table S4. Primer sequences used for qPCR (extracted from [7])

Target	Gen	Primer	Sequence (5'-3')	Amplicon size (bp)	Source
Buchnera aphidicola	*GroEL*	Forward	TCGGAAACGAAGCCCGAAT	204	[7]
		Reverse	CTTTTACCATTTGCGCACCC		
Regiella insecticola	*GyrB*	Forward	GAAGTGAAAACCGCCGTTGA	167	[7]
		Reverse	TCTAAAGCGCCTTTACGCCT		
Hamiltonella defensa	*GyrB*	Forward	CGCAAGGCAGTCATTATATTT TTG	201	[8]
		Reverse	GACAGATTTTTTGATATTCGC TACTTTG		
Elongation factor 1α	*EF-1α*	Forward	CTGATTGTGCCGTGCTTATTG	160	[9]
		Reverse	TATGGTGGTTCAGTAGAGTCC		

Table S5. Summary of evaluated traits across genotypes, facultative symbiont status, treatments, and generations. Survival is reported as the number of surviving individuals over the initial number of aphids, with the corresponding proportion in parentheses. Body mass and r_m are shown as mean ± SD. Dashes indicate cases in which the r_m value was not calculated

Genotype	Endosymbiont	Treatment	Generation	Survival ratio	Weight	r_m
Sa2	E-	control	G1	14/18 (0.78)	0.939 ± 0.251	0.264 ± 0.015
Sa2	E-	heat wave	G1	14/18 (0.78)	0.962 ± 0.196	0.265 ± 0.025
Sa2	E-	heat wave	G2	12/18 (0.67)	1.029 ± 0.175	---
Sa2	E+	control	G1	16/18 (0.89)	0.843 ± 0.218	0.268 ± 0.027
Sa2	E+	heat wave	G1	16/18 (0.89)	0.925 ± 0.213	0.275 ± 0.029
Sa2	E+	heat wave	G2	14/18 (0.78)	1.235 ± 0.258	---
Sa3	E-	control	G1	16/18 (0.89)	0.762 ± 0.231	0.252 ± 0.025

Genotype	Endosymbiont	Treatment	Generation	Survival ratio	Weight	r_m
Sa3	E-	heat wave	G1	13/18 (0.72)	0.720 ± 0.145	0.263 ± 0.025
Sa3	E-	heat wave	G2	12/18 (0.67)	0.891 ± 0.257	---
Sa3	E+	control	G1	17/18 (0.94)	0.802 ± 0.241	0.262 ± 0.028
Sa3	E+	heat wave	G1	13/18 (0.72)	0.776 ± 0.179	0.249 ± 0.017
Sa3	E+	heat wave	G2	13/18 (0.72)	1.039 ± 0.187	---
SaF16	E-	control	G1	14/18 (0.78)	0.820 ± 0.320	0.262 ± 0.028
SaF16	E-	heat wave	G1	16/18 (0.89)	0.746 ± 0.180	0.251 ± 0.029
SaF16	E-	heat wave	G2	15/18 (0.83)	0.929 ± 0.278	---
SaF16	E+	control	G1	23/36 (0.64)	0.733 ± 0.203	0.236 ± 0.039
SaF16	E+	heat wave	G1	17/36 (0.47)	0.679 ± 0.172	0.220 ± 0.030
SaF16	E+	heat wave	G2	11/36 (0.31)	0.669 ± 0.209	---

Table S6. ANOVA table for *survival*

Survival			
	LR c^2	df	*p*
genotype	1.695	2	0.428
symbiont	0.813	1	0.367
generation	0.556	1	0.455
genotype × symbiont	7.876	2	**0.019**
genotype × generation	0.083	2	0.958
symbiont × generation	0.049	1	0.824
genotype × symbiont × generation	0.152	2	0.926

Table S7. Pairwise contrasts for *survival*

Contrast	odds ratio	SE	z	*p*
Sa2-E- / Sa3-E-	1.144	0.594	0.259	1.000
Sa2-E- / SaF16-E-	0.419	0.255	-1.427	0.710

Sa2-E- / Sa2-E+	0.52	0.303	-1.124	0.872
Sa2-E- / Sa3-E+	1	0.526	0	1.000
Sa2-E- / SaF16-E+	4.086	1.81	3.172	**0.019**
Sa3-E- / SaF16-E-	0.367	0.221	-1.665	0.555
Sa3-E- / Sa2-E+	0.455	0.261	-1.371	0.745
Sa3-E- / Sa3-E+	0.874	0.454	-0.259	1.000
Sa3-E- / SaF16-E+	3.571	1.55	2.925	**0.040**
SaF16-E- / Sa2-E+	1.24	0.815	0.327	1.000
SaF16-E- / Sa3-E+	2.385	1.45	1.427	0.710
SaF16-E- / SaF16-E+	9.743	5.25	4.222	**<0.001**
Sa2-E+ / Sa3-E+	1.923	1.12	1.124	0.872
Sa2-E+ / SaF16-E+	7.857	3.99	4.055	**<0.001**
Sa3-E+ / SaF16-E+	4.086	1.81	3.172	**0.019**

Table S8. ANOVA table for *body mass*

Mb				
	SS	*F*	df	*p*
genotype	0.626	7.452	2	**<0.001**
symbiont	0.011	0.282	1	0.595
generation	0.033	0.803	1	0.371
genotype × symbiont	0.07	0.836	2	0.435
genotype × generation	0.062	0.746	2	0.475
symbiont × generation	0.222	5.300	1	**0.022**
genotype × symbiont × generation	0.367	4.371	2	**0.014**
residuals	7.186		171	

Table S9. Pairwise contrasts for *body mass*

Genotype	End	Contrast	Estimate	SE	t	*p*
Sa2	E-	G1 / G2	-0.067	0.075	-0.896	0.371
Sa3	E-	G1 / G2	-0.171	0.076	-2.266	**0.025**
SaF16	E-	G1 / G2	-0.183	0.072	-2.556	**0.011**
Sa2	E+	G1 / G2	-0.310	0.075	-4.152	**<0.001**
Sa3	E+	G1 / G2	-0.263	0.077	-3.436	**0.001**
SaF16	E+	G1 / G2	0.010	0.079	0.131	0.896

End	Generation	Contrast	Estimate	SE	t	*p*

E-	G1	Sa2 / Sa3	0.242	0.069	3.487	0.002
E-	G1	Sa2 / SaF16	0.216	0.068	3.160	0.005
E-	G1	Sa3 / SaF16	-0.026	0.069	-0.373	0.926
E+	G1	Sa2 / Sa3	0.149	0.070	2.115	0.090
E+	G1	Sa2 SaF16	0.247	0.068	3.607	0.001
E+	G1	Sa3 / SaF16	0.098	0.070	1.385	0.351
E-	G2	Sa2 / Sa3	0.138	0.080	1.710	0.204
E-	G2	Sa2 / SaF16	0.100	0.078	1.282	0.407
E-	G2	Sa3 / SaF16	-0.038	0.078	-0.488	0.877
E+	G2	Sa2 / Sa3	0.196	0.080	2.435	0.042
E+	G2	Sa2 / SaF16	0.567	0.084	6.746	**<0.001**
E+	G2	Sa3 / SaF16	0.371	0.084	4.415	**<0.001**

Genotype	Generation	Contrast	Estimate	SE	t	*p*
Sa2	G1	E- / E+	0.036	0.0683	0.532	0.596
Sa3	G1	E- / E+	-0.057	0.0714	-0.791	0.430
SaF16	G1	E- / E+	0.061	0.0683	0.979	0.329
Sa2	G2	E- / E+	-0.207	0.0804	-2.570	**0.011**
Sa3	G2	E- / E+	-0.148	0.0804	-1.845	0.067
SaF16	G2	E- / E+	0.260	0.0814	3.199	**0.002**

Table S10. ANOVA table for *fecundity*

fecundity			
	LR c^2	df	*p*
genotype	7.122	2	**0.028**
symbiont	0.101	1	0.749
treatment	0.291	1	0.589
genotype × symbiont	18.859	2	**<0.001**
genotype × treatment	5.912	2	0.052
symbiont × treatment	1.499	1	0.22
genotype × symbiont × treatment	29.172	2	**<0.001**

Table S11. Pairwise contrasts for *fecundity*

Genotype	Trat	Contrast	Estimate	SE	z	p
Sa2	Control	E- / E+	-0.002	0.070	-0.319	0.750
Sa3	Control	E- / E+	-0.121	0.076	-1.592	0.112

SaF16	Control	E- / E+	0.308	0.072	4.306	**<0.001**
Sa2	Treatment	E- / E+	-0.014	0.071	-2.046	**0.041**
Sa3	Treatment	E- / E+	-0.066	0.081	-0.815	0.415
SaF16	Treatment	E- / E+	0.947	0.078	12.042	**<0.001**

Genotype	End	Contrast	Estimate	SE	z	p
Sa2	E-	Control / Treatment	0.039	0.072	0.539	0.590
Sa3	E-	Control / Treatment	0.146	0.081	1.798	0.072
SaF16	E-	Control / Treatment	-0.121	0.076	-1.589	0.112
Sa2	E+	Control / Treatment	-0.084	0.070	-1.202	0.230
Sa3	E+	Control / Treatment	0.200	0.077	2.62	0.009
SaF16	E+	Control / Treatment	0.519	0.075	6.954	**<0.001**

End	Trat	Contrast	Estimate	SE	z	p
E-	Control	Sa2 / Sa3	0.157	0.075	2.085	0.093
E-	Control	Sa2 / SaF16	0.183	0.075	2.453	**0.0377**
E-	Control	Sa3 / SaF16	0.026	0.079	0.333	0.941
E+	Control	Sa2 / Sa3	0.059	0.072	0.822	0.690
E+	Control	Sa2 SaF16	0.514	0.068	7.617	**<0.001**
E+	Control	Sa3 / SaF16	0.455	0.069	6.638	**<0.001**
E-	Treatment	Sa2 / Sa3	0.264	0.078	3.39	**0.002**
E-	Treatment	Sa2 / SaF16	0.024	0.073	0.329	0.942
E-	Treatment	Sa3 / SaF16	-0.240	0.078	-3.065	**0.006**
E+	Treatment	Sa2 / Sa3	0.343	0.075	4.574	**<0.001**
E+	Treatment	Sa2 / SaF16	1.167	0.077	14.539	**<0.001**
E+	Treatment	Sa3 / SaF16	0.774	0.082	9.45	**<0.001**

Table S12. ANOVA table for *intrinsic rate of population increase (r_m)*

r_m

	SS	*F*	df	*p*
genotype	0.001	0.805	2	0.448
symbiont	<0.001	0.189	1	0.664
treatment	<0.001	0.01	1	0.919
genotype × symbiont	0.006	3.973	2	**0.02**
genotype × treatment	0.001	1.065	2	0.346
symbiont × treatment	<0.001	0.134	1	0.714
genotype × symbiont ×treatment	0.001	1.048	2	0.352
residuals	0.137		176	

Table S13. Pairwise contrasts for *Intrinsic population growth rate* (r_m)

Genotype	Trat	Contrast	Estimate	SE	t	p
Sa2	Control	E- / E+	-0.0045	0.010	-0.435	0.664
Sa3	Control	E- / E+	-0.0103	0.010	-1.059	0.291
SaF16	Control	E- / E+	0.0254	0.009	2.682	**0.008**
Sa2	Treatment	E- / E+	-0.0098	0.010	-0.954	0.342
Sa3	Treatment	E- / E+	0.0141	0.011	1.26	0.209
SaF16	Treatment	E- / E+	0.0312	0.010	3.206	**0.002**

Genotype	End	Contrast	Estimate	SE	t	p
Sa2	E-	Control / Treatment	-0.0011	0.011	-0.101	0.919
Sa3	E-	Control / Treatment	-0.0110	0.010	-1.058	0.292
SaF16	E-	Control / Treatment	0.0103	0.010	1.003	0.317
Sa2	E+	Control / Treatment	-0.0064	0.010	-0.645	0.520
Sa3	E+	Control / Treatment	0.0134	0.011	1.269	0.206
SaF16	E+	Control / Treatment	0.0161	0.009	1.797	0.074

End	Trat	Contrast	Estimate	SE	t	p
E-	Control	Sa2 / Sa3	0.0121	0.010	1.185	0.464
E-	Control	Sa2 / SaF16	0.0024	0.011	0.223	0.973
E-	Control	Sa3 / SaF16	-0.0098	0.010	-0.955	0.607
E+	Control	Sa2 / Sa3	0.0063	0.010	0.643	0.797
E+	Control	Sa2 SaF16	0.0322	0.009	3.541	**0.002**
E+	Control	Sa3 / SaF16	0.0259	0.009	2.903	**0.012**
E-	Treatment	Sa2 / Sa3	0.0022	0.011	0.2	0.978
E-	Treatment	Sa2 / SaF16	0.0137	0.010	1.339	0.376
E-	Treatment	Sa3 / SaF16	0.0115	0.010	1.106	0.512
E+	Treatment	Sa2 / Sa3	0.0260	0.011	2.437	**0.042**
E+	Treatment	Sa2 / SaF16	0.0546	0.010	5.614	**<0.001**
E+	Treatment	Sa3 / SaF16	0.0286	0.011	2.718	**0.020**

Table S14. ANOVA table for *B. aphidicola* titer (full model) and individual ANOVA models within genotype

Buchnera aphidicola titer				
source	SS	df	*F*	*p*
genotype	0.754	2	1.822	0.183
symbiont	0.78	1	3.766	0.064
treatment	0.168	1	0.815	0.375
genotype × symbiont	1.562	2	3.772	**0.037**
genotype × treatment	0.107	2	0.258	0.774
symbiont × treatment	0.002	1	0.011	0.916
genotype × symbiont ×treatment	0.342	2	0.826	0.449
residuals	4.969	24		

Sa2	source	SS	df	*F*	*p*
	treatment	<0.001	1	<0.001	0.993
	symbiont	0.054	1	0.583	0.466
	interaction	<0.001	1	0.005	0.941
	residuals	0.751	8		
Sa3	source	SS	df	*F*	*p*
	treatment	0.211	1	1.074	0.33
	symbiont	0.041	1	0.21	0.658
	interaction	0.196	1	1.002	0.346
	residuals	1.570	8		
SaF16	source	SS	df	*F*	*p*
	treatment	0.065	1	0.196	0.669
	symbiont	2.246	1	6.787	**0.031**
	interaction	0.147	1	0.445	0.523
	residuals	2.647	8		

Table S15. ANOVA table for the facultative symbiont titer within genotype

Sa2	source	SS	df	*F*	*p*
	treatment	0.016	1	0.389	0.55
	generation	<0.001	1	<0.001	0.999
	interaction	0.491	1	1.192	0.306
	residuals	0.329	8		

Sa3	source	SS	df	*F*	*p*
	treatment	0.029	1	1.163	0.312
	generation	<0.001	1	<0.001	0.997
	interaction	<0.001	1	0.019	0.892
	residuals	0.202	8		

SaF16	source	SS	df	*F*	*p*
	treatment	0.092	1	0.394	0.547
	generation	<0.001	1	<0.001	0.999
	interaction	0.541	1	0.231	0.643
	residuals	1.874	8		

Table S16. Results of the simulation-based post-hoc power analyses

Survival

Genotype	Generation	Contrast	power
Sa2	G1	E- / E+	0.043
Sa2	G2	E- / E+	0.083
Sa3	G1	E- / E+	0.041
Sa3	G2	E- / E+	0.067
SaF16	G1	E- / E+	0.77
SaF16	G2	E- / E+	0.0935

Body mass

Genotype	Generation	Contrast	power
Sa2	G1	E- / E+	0.031
Sa2	G2	E- / E+	0.074
Sa3	G1	E- / E+	0.064
Sa3	G2	E- / E+	0.1
SaF16	G1	E- / E+	0.022
SaF16	G2	E- / E+	0.463

Fecundity

Genotype	Treatment	Contrast	power
Sa2	control	E- / E+	0.044
Sa2	heat wave	E- / E+	0.216
Sa3	control	E- / E+	0.336
Sa3	heat wave	E- / E+	0.175
SaF16	control	E- / E+	0.97
SaF16	heat wave	E- / E+	0.99

r_m

Genotype	Treatment	Contrast	power
Sa2	control	E- / E+	0.056
Sa2	heat wave	E- / E+	0.033
Sa3	control	E- / E+	0.077
Sa3	heat wave	E- / E+	0.035
SaF16	control	E- / E+	0.044
SaF16	heat wave	E- / E+	0.099

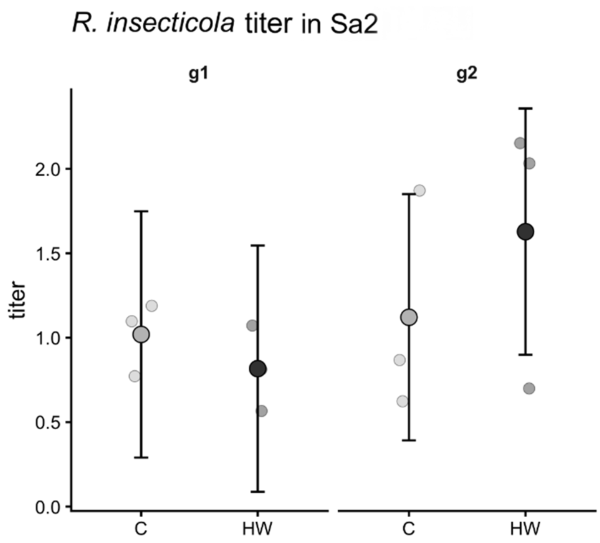

Figure S1: Relative titer of *R. insecticola* in genotype Sa2 from *S. avenae* under Control (C) and heat wave (HW) treatments and generations (g1 and g2). Values represent estimated marginal means (±95% CI) derived from lineage-specific analyses.

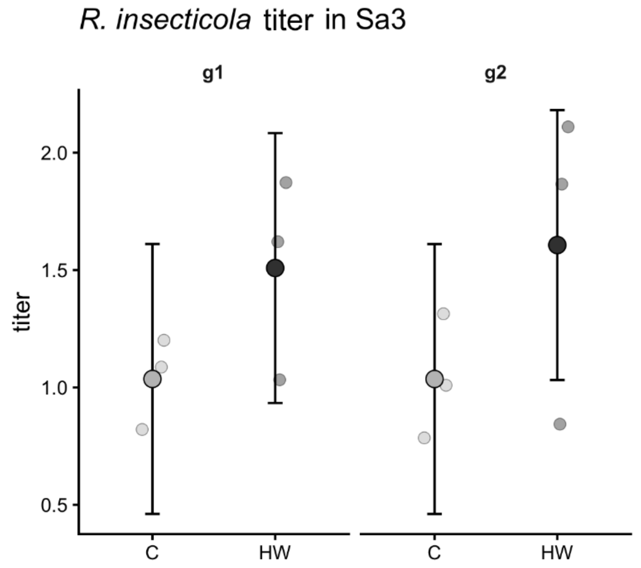

Figure S2: Relative titer of *R. insecticola* in genotype Sa3 from *S. avenae* under Control (C) and heat wave (HW) treatments and generations (g1 and g2). Values represent estimated marginal means (±95% CI) derived from lineage-specific analyses.

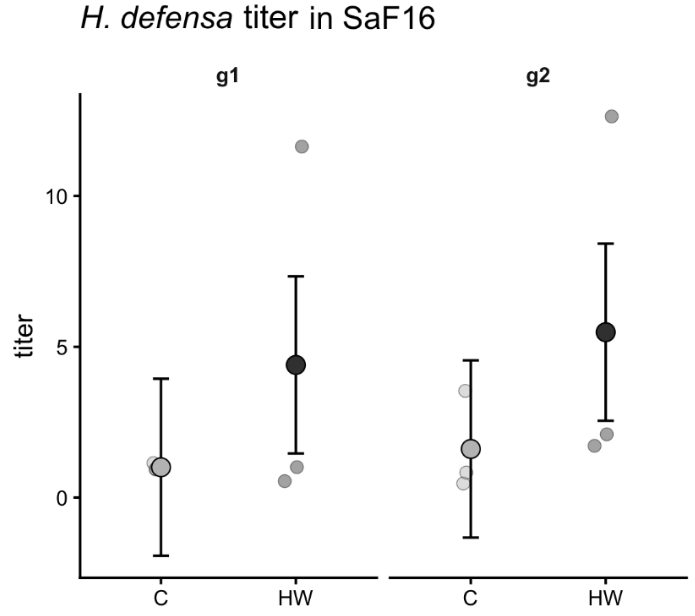

Figure S3: Relative titer of *H. defensa* in genotype SaF16 from *S. avenae* under Control (C) and heat wave (HW) treatments and generations (g1 and g2). Values represent estimated marginal means (±95% CI) derived from lineage-specific analyses.

References

1. Llewellyn, K.S.; Loxdale, H.D.; Harrington, R.; Brookes, C.P.; Clark, S.J.; Sunnucks, P. Migration and Genetic Structure of the Grain Aphid (Sitobion Avenae) in Britain Related to Climate and Clonal Fluctuation as Revealed Using Microsatellites. *Mol. Ecol.* **2003**, *12*, 21–34, doi:10.1046/J.1365-294x.2003.01703.x.
2. Wilson, A.; Massonnet, B.; Simon, J.C.; Prunier-Leterme, N.; Dolatti, L.; Llewellyn, K.S.; Figueroa, C.C.; Ramírez, C.C.; Blackman, R.L.; Estoup, A.; et al. Cross-Species Amplification of Microsatellite Loci in Aphids: Assessment and Application. *Mol. Ecol. Notes* **2004**, *4*, 104–109, doi:10.1046/j.1471-8286.2003.00584.x.
3. Leonardo, T.E. Removal of a Specialization-Associated Symbiont Does Not Affect Aphid Fitness. *Ecol. Lett.* **2004**, *7*, 461–468, doi:10.1111/J.1461-0248.2004.00602.X.
4. Peccoud, J.; Bonhomme, J.; Mahéo, F.; de la Huerta, M.; Cosson, O.; Simon, J.-C. Inheritance Patterns of Secondary Symbionts during Sexual Reproduction of Pea Aphid Biotypes. *Insect Sci.* **2013**, *21*, 291–300, doi:10.1111/1744-7917.12083.
5. Tsuchida, T.; Koga, R.; Horikawa, M.; Tsunoda, T.; Maoka, T.; Matsumoto, S.; Simon, J.-C.; Fukatsu, T. Symbiotic Bacterium Modifies Aphid Body Color. *Science* **2010**, *330*, 1102–1104, doi:10.1126/science.1195463.
6. Guay, J.-F.; Boudreault, S.; Michaud, D.; Cloutier, C. Impact of Environmental Stress on Aphid Clonal Resistance to Parasitoids: Role of *Hamiltonella Defensa* Bacterial Symbiosis in Association with a New Facultative Symbiont of the Pea Aphid. *J. Insect Physiol.* **2009**, *55*, 919–926, doi:10.1016/j.jinsphys.2009.06.006.
7. Díaz-Hernández, A.M.; Sepúlveda, D.A.; González-González, A.; Briones, L.M.; Correa, M.C.G.; Figueroa, C.C. Water Deficit and Aphid Resilience on Wheat: Examining *Sitobion Avenae* F. and Their Bacterial Symbionts Interplay under Controlled Laboratory Conditions. *Pest Manag. Sci.* **2024**, doi:10.1002/PS.8428.
8. Clarke, H.V. Genotypic and Endosymbiont-Mediated Variation in Parasitoid Susceptibility and Other Fitness Traits of the Potato Aphid, *Macrosiphum Euphorbiae*., University of Dundee: Dunde, 2013.
9. Oliver, K.M.; Russell, J.A.; Moran, N.A.; Hunter, M.S. Facultative Bacterial Symbionts in Aphids Confer Resistance to Parasitic Wasps. *Proc. Natl. Acad. Sci. U. S. A.* **2003**, *100*, 1803–1807, doi:10.1073/pnas.0335320100.
